# Synergistic effect of composite microbial bioactive metabolites in the control of rice diseases and pest insects under reduced-dosage chemical pesticide application

**DOI:** 10.3389/fmicb.2026.1771704

**Published:** 2026-06-15

**Authors:** Zhenxu Bai, Lusheng Chen, Cheng Zhang, Jingyi Liu, Bo Lang, Xifen Zhang, Hongyi Liu, Jie Chen

**Affiliations:** 1 School of Agriculture and Biology, Shanghai Jiao Tong University, Shanghai, China; 2 Shanghai Yangtze River Delta Eco-Environmental Change and Management Observation and Research Station, Ministry of Science and Technology, Ministry of Education, Shanghai, China; 3 State Key Laboratory of Microbial Metabolism, Shanghai Jiao Tong University, Shanghai, China

**Keywords:** biological control, composite microbial metabolites, reduced chemical pesticides, rice diseases and pests, synergistic control

## Abstract

To advance green rice protection strategies, this study developed a novel microbial bioactive formulation (TMB) from composite metabolites of *Trichoderma asperellum*, *Metarhizium anisopliae*, and *Bacillus subtilis*, and systematically evaluated its synergy with reduced-dosage chemical pesticides against rice disease and pests. In vitro antifungal assays identified the optimal TMB volume ratio as 1:1:1 (v/v/v), which achieved 85.3 and 67.4% inhibition rates against *Curvularia plantarum* and *Fusarium fujikuroi*, respectively, significantly higher than single-strain metabolites (*p* < 0.05). The biochar-based TMB seed coating agent suppressed *Rhizoctonia solani*- induced rice sheath blight by 75.56%, and significantly improved rice seed germination under *C. plantarum* and *F. fujikuroi* co-infection (*p* < 0.05). The co-application of TMB with 30–50% reduced dosage of 75% trifloxystrobin tebuconazole resulted in a higher inhibition rate against *C. plantarum* than the equivalent dosage of chemical pesticide alone, reaching a control level comparable to that of full-dose chemical pesticide. Field trials demonstrated that TMB combined with 30–40% reduced chemical pesticides achieved excellent synergistic control effects against rice sheath blight, rice spikelet rot, rice grain black spot, and key rice pest insects including the brown planthopper (*Nilaparvata lugens*), and rice leaf roller (*Cnaphalocrocis medinalis*), with control efficacy generally reaching or surpassing that of full-dose chemical treatments. Specifically, TMB combined with a 40% reduction in chemical pesticides achieved over 50% control efficacy against *N. lugens* and over 75% against *C. medinalis*; Compared with full-dose chemicals, the treatment with TMB combined with a 30% reduction in tebuconazole⋅azoxystrobin reduced the incidence of rice grain black spot by more than 60%. Notably, we identified multiple antifungal, insecticidal and plant growth-promoting bioactive components in the TMB formulation, and confirmed that the biochar carrier can effectively adsorb and protect these active compounds, significantly enhancing the storage stability of the formulation. This finding lays a material foundation for the quality control and standardized production of the TMB-based biocontrol agent. This study confirms that the synergistic application of TMB with reduced-dosage chemical pesticides provides a feasible and effective strategy for efficient green control of rice diseases and pest insects, while achieving a 30% reduction in chemical pesticide inputs in rice production.

## 1 Introduction

Rice is one of the most important staple crops worldwide, and its stable production is critical to global food security. However, rice production is severely threatened by multiple diseases and insect pests, including rice blast, rice sheath blight, rice spikelet rot, rice grain black spot, *Cnaphalocrocis medinalis*, *Chilo suppressalis*, and *Nilaparvata lugens* (Deutsch et al., 2018; Gurr et al., 2016; Hu et al., 2023; Shrivastava and Pradhan, 2020). Although the global incidence of rice blast has been effectively controlled through the breeding and popularization of resistant cultivars targeting *Magnaporthe oryzae* (Devanna et al., 2022), recent climate anomalies have led to frequent outbreaks of previously minor rice diseases and pests, causing substantial yield losses (Savary et al., 2019). For example, severe outbreaks of rice grain black spot and spikelet rot caused by *Curvularia plantarum* in Songjiang District, Shanghai, China, resulted in a 26.77% yield loss in local rice production (Bai et al., 2024).

At present, the control of rice diseases and insect pests still relies heavily on chemical pesticides (Singh et al., 2019). However, long-term and excessive application of chemical pesticides, even low-toxicity varieties, poses multiple risks: it drives the development of pesticide resistance in pathogen and pests, causes environmental pollution and pesticide residue accumulation in agricultural products, and disrupts the structure of beneficial soil microbiota and natural enemy biodiversity, ultimately wakening the intrinsic pest regulation capacity of farmland ecosystems (Yadav et al., 2015; Md Meftaul et al., 2020; Hilliou et al., 2021; Senapati et al., 2022; Narayanan et al., 2024). Therefore, there is an urgent need to develop novel biocontrol technologies to reduce chemical pesticide inputs, while ensuring efficient pest control and maintaining the health of agroecosystems.

*Trichoderma* spp. and *Bacillus* spp. are the most widely used biocontrol microorganisms worldwide, and their live strains and secondary metabolites are the core components of most commercial bio-pesticide (Kaspar et al., 2019; Zin and Badaluddin, 2020). The entomopathogenic fungus *Metarhizium anisopliae* is also extensively applied for the control of agricultural insect pests (Beys-da-Silva et al., 2020). Previous studies have shown that microbial consortia integrating these biocontrol agents can achieve synergistic enhancement of control efficacy (Dai et al., 2022; Wu et al., 2023). However, live microbial biocontrol agents have critical limitations in practical application, including poor storage stability, short shelf-life, susceptibility to environmental stress, and incompatibility with chemical pesticides (Guzmán-Guzmán et al., 2019; Köhl et al., 2019; Tyśkiewicz et al., 2022).

In contrast, microbial secondary metabolites have unique advantages: longer shelf stability, rapid onset of action, and high compatibility with synthetic chemical pesticides, representing a next-generation strategy for green plant protection. However, current applications are largely limited to single-strain metabolites with narrow pest spectra, failing to address the complex disease–pest complexes in rice production. Previous studies have confirmed that *Trichoderma* spp. metabolites contain antifungal and plant beneficial active substances such as peptaibols, 6-Pentyl-α-pyrone (Mukherjee et al., 2012), *Bacillus* spp. secrete antimicrobial lipopeptides including surfactin, fengycin, and kurstakin (Ongena and Jacques, 2008), and *M. anisopliae* produces insecticidal toxins and lipases with insecticidal and antifungal activities (Kershaw et al., 1999; Beys da Silva et al., 2010). We therefore hypothesize that a ternary composite metabolite system (TMB) integrating complementary functional metabolites from *T. asperellum* (antifungal and resistance-inducing), *M. anisopliae* (insecticidal and dual antifungal), and *B. subtilis* (broad-spectrum antagonistic) can exert broad-spectrum, synergistic biocontrol activity against multiple rice pathogens and insect pests simultaneously. Furthermore, we hypothesize that this TMB formulation can act synergistically with reduced doses of chemical pesticides, allowing a certain reduction in chemical inputs while maintaining or enhancing control efficacy against major threats such as *Rhizoctonia solani*, *Fusarium fujikuroi*, *Curvularia plantarum*, *Cnaphalocrocis medinalis*, and *Nilaparvata lugens*. This synergistic system is expected to mitigate pathogen/pest resistance, lower environmental risks, and establish a feasible model for integrated, chemical-reducing rice health management.

To test the above hypotheses and fill the critical knowledge gaps in cell-free microbial metabolite-based integrated pest management for rice production, we defined four core research objectives in this study. First, we aim to optimize the volume ratio of fermentation metabolites from *T. asperellum*, *M. anisopliae*, and *B. subtilis* via in vitro antifungal assays against the key rice pathogenic fungi *C. plantarum* and *F. fujikuroi*, to develop a broad-spectrum composite microbial metabolite formulation (TMB). Second, we seek to systematically evaluate the synergistic interaction between TMB and mainstream chemical pesticides, and to clarify the synergistic control potential of their combined application under 30–50% dosage reduction of chemical inputs. Third, we intend to develop a biochar-based TMB seed coating agent, and to investigate its suppressive effect on seed-borne *C. plantarum* and *F. fujikuroi*, as well as its impact on rice seed germination and seedling growth. Finally, we aim to verify the synergistic control efficacy of TMB combined with reduced-dosage chemical pesticides against multiple rice diseases and insect pests through multi-site field trials, to provide a robust scientific basis and practical technical protocol for establishing a green, efficient, and chemical-reducing integrated management strategy for rice health in sustainable agricultural systems.

## 2 Materials and methods

### 2.1 Tested strains

*Trichoderma asperellum* CTCCSJ-W-SBW10264, *Bacillus subtilis* S4-4-10, *Metarhizium anisopliae* CGMCC No. 3.11962, *Rhizoctonia solani*, *Curvularia plantarum*, and *Fusarium fujikuroi* were maintained at the *Trichoderma* Culture Collection Center of Shanghai Jiao Tong University, Shanghai, China.

### 2.2 Preparation of composite metabolites

For filamentous fungi (*T. asperellum* and *M. anisopliae*): Strains were first activated and cultured on potato dextrose agar (PDA) plates (200 g potato, 20 g glucose, 20 g agar, distilled water to 1 L, pH 5.6) at 28 °C for 7 days. Conidia on the plates were rinsed down using sterile 0.1% Tween 80 solution to prepare a spore suspension, which was adjusted to a concentration of 1 × 10^6^ CFU/mL with a hemocytometer for subsequent inoculation. For *B. subtilis*: The strain was activated and cultured on Luria-Bertani (LB) agar plates (10 g tryptone, 5 g yeast extract, 10 g NaCl, 20 g agar, distilled water to 1 L, pH 7.4) at 28 °C for 24 h. A single colony was picked and inoculated into LB liquid medium, followed by shaking incubation at 28 °C and 180 rpm for 12 h to prepare a seed suspension. The optical density was adjusted to OD_600_ = 0.8 for inoculation.

Fungal fermentation: 1 mL of spore suspension (1 × 10^6^ CFU/mL) of *T. asperellum* or *M. anisopliae* was inoculated into 250 mL Erlenmeyer flasks containing 100 mL potato dextrose (PD) broth. Bacterial fermentation: 1 mL of *B. subtilis* suspension (OD_600_ = 0.8) was inoculated into PD broth under the same conditions. All cultures were incubated in the dark at 28°C with shaking at 180 rpm for 5 days. The fermentation supernatants (containing extracellular metabolites) were harvested by centrifugation at 12,000 × g for 10 min at 4°C, followed by sterile filtration through 0.22 μm pore-size filters. The composite metabolites were prepared by mixing the sterile-filtered metabolites of *T. asperellum*, *M. anisopliae*, and *B. subtilis* at the designated ratios, and the resulting composite metabolites agent was designated TMB.

### 2.3 Inhibitory effects of composite microbial metabolites on rice pathogenic fungi

Plate preparation Metabolite solutions, including single-strain metabolites of *T. asperellum*, *M. anisopliae*, and *B. subtilis*, as well as composite metabolites TMB at a 1:1:1 ratio, were mixed with molten potato dextrose agar (PDA) medium at a 1:3 (v/v) ratio. The mixture was poured into sterile Petri dishes and allowed to solidify at room temperature. A 5 mm diameter mycelial plug of the target pathogen (*F. fujikuroi* or *C. plantarum*), cut from the leading edge of an actively growing PDA colony using a sterile cork borer, was inoculated upside down onto the center of each plate. Control plates were prepared by mixing sterile PD broth with PDA at the same 1:3 (v/v) ratio to match the dilution and PD content of the metabolite-treated groups. All plates were incubated at 28°C in the dark. When the mycelial colony in the control plates covered two-thirds of the plate diameter, the colony diameter of each plate were measured using the cross method. The mycelial growth inhibition rate was calculated using the following formula: Inhibition rate (%) = [(Control colony radius - Treatment colony radius)/Control colony radius] × 100. Ratio optimization assay: The ratio of TMB was optimized by adjusting the proportion of *B. subtilis* metabolite (T:M:B = 1:1:2, 1:1:3, 1:1:4, 1:1:5). Dilution series assay: TMB was mixed with molten PDA at ratios of 1:4, 1:9, 1:14, 1:19, and 1:29 (equivalent to 4×, 10×, 15×, 20×, and 30× dilutions, respectively). After solidification, the plates were inoculated with *C. plantarum* or *F. fujikuroi* as described above. Control plates were prepared with PDA containing the corresponding volume of sterile PD broth. Colony diameter measurement and inhibition rate calculation followed the same protocol described above. All assays were performed in triplicate.

### 2.4 Control efficacy of composite metabolites against rice spikelet rot disease

Rice plants of the *Nangeng 46* cultivar were grown in four experimental plots, and foliar sprays were applied respectively as follows: sterile water (control), 50-fold diluted TMB (TMB-50 × ), 100-fold diluted TMB (TMB-100 × ), and 200-fold diluted TMB (TMB-200 × ). At 72 h after spraying, all plots were inoculated with a *C. plantarum* spore suspension (1 × 10^6^ CFU/mL containing 0.2% Tween 20) via foliar spraying, with concurrent stem injection performed on at least 5 hills per plot (2 mL of the same spore suspension was injected into 10 plants per hill).

### 2.5 Synergistic inhibitory effect of composite metabolites and chemical fungicides on rice pathogenic fungi

Two chemical fungicides, 29% trifloxystrobin⋅tebuconazole and 75% tebuconazole⋅azoxystrobin, were selected for the synergistic assay. Serial dilutions of each fungicide (1, 2, 4, 8, 16, 32, and 64 mg/L) were prepared to determine the median effective concentration (EC_50_) values against *C. plantarum* and *F. fujikuroi*.

TMB composite metabolites (1:1:1 ratio) were diluted 30-fold, then mixed with the fungicide dose corresponding to 50% mycelial growth inhibition of the target pathogens. The mixtures were incorporated into molten PDA medium, poured into plates, and inoculated with a 5 mm mycelial plug of *C. plantarum* or *F. fujikuroi* as described in section 2.3. Control plates were prepared with PDA mixed with 30% (v/v) sterile PD broth. All plates were incubated at 28°C, and colony radii were measured when the control colonies covered two-thirds of the plate diameter. The inhibition rate was calculated using the formula described in section 2.3.

For the reduced-dose chemical synergy assays, the field-recommended doses of the fungicides were reduced by 30, 40, and 50%, respectively, before mixing with 30-fold diluted TMB. The plating, inoculation, and measurement protocols were identical to those described above.

### 2.6 Preparation of biochar-Based TMB seed coating agent and its effects on pathogen suppression and rice growth

Preparation of TMB seed coating agent using the sustained-release properties of biochar: 0.6 g of biochar (Rice straw was pyrolyzed in a muffle furnace at 400°C for 2 h to obtain the biocha), 0.3% (w/v) sodium carboxymethyl cellulose (CMC-Na), and 2 mL of TMB (the three microbial metabolite components at a ratio of 1:1:1) were mixed, and brought to a final volume of 20 mL with sterile water. The mixture was homogenized at 200 rpm for 30 min at room temperature to obtain a uniform and stable biochar-based TMB seed coating agent. The physicochemical properties of the prepared seed coating agent were characterized as follows: viscosity of 350–400 mPa⋅s, film formation time of 15–20 min at room temperature, coating uniformity ≥ 95%, and coating shedding rate ≤ 5%. To evaluate the suppression of *C. plantarum* and *F. fujikuroi* infection, preventive and curative treatment groups were set up: Preventive group: Rice seeds were immersed in the biochar-TMB coating agent for 24 h, followed by 24 h exposure to the pathogen spore suspension (1 × 106 CFU/mL); Curative group: Rice seeds were first exposed to the pathogen spore suspension for 24 h, then immersed in the biochar-TMB coating agent for 24 h; Control groups: Seeds treated with biochar coatings loaded with single-strain metabolites, and blank biochar coating without TMB. Thirty seeds per treatment (with three biological replicates) were placed for germination on moist sterile filter paper. Germination rate, root length, and shoot length were recorded after 5 days of incubation at 28 °C in the dark. For the rice sheath blight control assay, rice seeds were surface-sterilized with 75% (v/v) ethanol for 5 min, treated with biochar-TMB for 48 h, germinated for 5 days, and transplanted into pots filled with soil amended with 3–5% (w/w) *R. solani* or *F. fujikuroi*-infested wheat grains. After 14 days of growth, plant height was measured, and the disease index was assessed using a 0–9 rating scale to calculate the control efficacy.

Fourier Transform Infrared Spectroscopy (FTIR) and Scanning Electron Microscopy (SEM) Analysis: Biochar control and biochar-TMB mixtures stored at 4 °C, room temperature, and 50 °C for 30 days were centrifuged to separate the solid biochar fraction, followed by vacuum freeze-drying. FTIR analysis was performed in the 2,000–400 cm^–1^ wavenumber range to characterize the functional groups of bioactive compounds adsorbed by biochar, and SEM was used to observe the microscopic morphological changes of the biochar samples after storage. High-Performance Liquid Chromatography-Tandem Mass Spectrometry (HPLC-MS) Targeted Detection The original TMB solution and biochar-TMB mixtures stored at different temperatures for 30 days were extracted with an equal volume of ethyl acetate. The organic phase was collected, dried under nitrogen flow, and redissolved in chromatographic grade methanol. Targeted analysis of the bioactive marker compounds was conducted using an ultra-performance liquid chromatography-ion mobility-quadrupole time-of-flight mass spectrometer (VION, Waters).

### 2.7 Field demonstration of composite metabolites for rice disease and pest control

#### 2.7.1 Trial sites and rice varieties

To validate the broad applicability of our findings, field trials were carried out in 2023 at two major rice-producing districts in Shanghai, China: Jinshan (121°00’E, 30°40’N; 1.17 hm^2^, rice cultivar *Nangeng 46*, seeding rate 6 kg/hm^2^) and Songjiang (121°13’E, 31°01’N; 1.33 hm^2^, rice cultivar *Song 1013*, seeding rate 6 kg/hm^2^). Both sites followed conventional local rice cultivation practices, while differing significantly in agro-environmental conditions, pest and disease spectrums, and local pesticide application regimes. Specifically, rice sheath blight, rice spikelet rot, *C. medinalis* and *N. lugens* were monitored in Jinshan District, with rice sheath blight, rice spikelet rot and rice grain black spot as the core targets in Songjiang District.

#### 2.7.2 Trial treatments and experimental design

The chemical pesticides used at the trial site in Jinshan District, Shanghai are detailed in Table 1; all pesticides used in Jinshan District were supplied by the local government. At the trial site in Songjiang District, Shanghai, the following commercial pesticides were used: 29% trifloxystrobin⋅tebuconazole water-dispersible granules (WG, purchased from Bayer), and 75% tebuconazole⋅azoxystrobin suspension concentrate (SC, purchased from Nanjing Huiyu Agrochemical Co., Ltd.). Additional auxiliary agents in Songjiang District were supplied by the local agricultural extension centers. All pesticides were applied at the manufacturer-recommended rates with a spray volume of 750 L per hectare.

**TABLE 1:** Chemical pesticides applied in Jinshan District, Shanghai.

Rice growth stage	Pesticide name	Formulation	Rate (/hm^2^)	Target pests/diseases
Tillering	40% chlorantraniliprole ⋅ thiamethoxam	WG	150 g	*C. medinalis*, *N. lugens*
	75% tebuconazole ⋅ azoxystrobin	WG	225 g	Rice Sheath Blight
Jointing	23% kresoxim-methyl ⋅ epoxiconazole	SC	600 g	Rice Sheath Blight
	16% emamectin benzoate ⋅ indoxacarb	SC	225 g	*C. medinalis*
Initial heading	75% tricyclazole	WP	450 g	Rice Blast
	25% methoxyfenozide ⋅ indoxacarb	SC	450 g	*C. medinalis*
	24% validamycin A	AS	600 g	Rice Sheath Blight, Rice False smut
	50% pymetrozine	WP	225 g	*N. lugens*
Late heading	20% chlorantraniliprole ⋅ fluxapyroxad	SC	150 g	*C. medinalis*
	29% trifloxystrobin ⋅ tebuconazole	WG	225 g	Rice Sheath Blight, Rice False smut

WG, water-dispersible granules; SC, suspension concentrate; WP, wet table powder; AS, aqueous solution.

At the Jinshan District site (targeting Rice Sheath Blight, Rice Spikelet Rot Disease, *C. medinalis*, and *N. lugens*), three treatments were set up: Treatment 1 (two replicates, 0.163 hm^2^ per replicate) : Seed coating with biochar-TMB plus a 30% reduced dosage of 32% tebuconazole⋅ethylicin, soil amendment with *Trichoderma* granules (Gaolenian^®^, 75 kg/hm^2^) as basal fertilizer, foliar sprays of TMB plus chemical pesticides (Table 1) at a 40% reduced dosage. Treatment 2 (two replicates, 0.332 hm^2^ per replicate): Seed treatment with the full recommended dose of 32% tebuconazole⋅ethylicin (31 g/hm^2^), soil amendment with *Trichoderma* granules, foliar sprays of full-dose chemical sprays (Table 1). Untreated control (two replicates, 0.092 hm^2^ per replicate): Identical seed/soil treatment as the above groups, with no foliar pesticide or TMB applications.

At the Songjiang District site (targeting Rice Sheath Blight, Rice Spikelet Rot Disease, and Rice Black Spot), five treatments were set up (two replicates, 0.13 hm^2^ per replicate). All treatments shared the same baseline practices: seed treatment with 32% tebuconazole⋅ethylicin suspension, soil amendment with *Trichoderma* granules, and full-dose insecticide sprays (Table 2). The specific foliar fungicide treatments were as follows: Treatment 1: 30% reduced dosage of 29% trifloxystrobin⋅tebuconazole WG plus TMB, applied at the initial and late booting stages; Treatment 2: 30% reduced dosage of 75% tebuconazole⋅azoxystrobin WG plus TMB, applied at the booting stages; Treatment 3: Full recommended dosage of 29% trifloxystrobin⋅tebuconazole (195 g/hm^2^), applied at the booting stages; Treatment 4: Full recommended dosage of 75% tebuconazole azoxystrobin (225 g/hm^2^), applied at the booting stages; Untreated Control: No foliar fungicide applications. Notably, full-dose insecticides listed in Table 2 were consistently applied across all treatments in Songjiang District to ensure uniform pest control.

**TABLE 2:** Summary of chemical insecticides used in Songjiang District, Shanghai.

Rice growth stage	Pesticide name	Formulation	Rate (/hm^2^)	Target pests
Initial heading	50% pymetrozine water-dispersible granules	WG	300 g	*N. lugens*
	25% methoxyfenozide ⋅ indoxacarb suspension concentrate	SC	225 g	*C. medinalis*
Late heading	25% methoxyfenozide ⋅ indoxacarb suspension concentrate	SC	120 g	*C. medinalis*
	50% nitenpyram water-dispersible granules	WG	120 g	*N. lugens*

WG, water-dispersible granules; SC, suspension concentrate.

#### 2.7.3 Efficacy assessment

##### 2.7.3.1 Disease efficacy assessment

Disease indices and control efficacy for rice sheath blight and rice spikelet rot were assessed following Qin et al. (2013) and Chen J. et al. (2025). Disease and pest incidence was evaluated 6 days post-application. Control efficacy (%) calculated as: [(Disease Index of control- Disease Index of treatment) / Disease Index of control] × 100. For Rice Black Spot, 100 randomly selected grains per treatment (triplicate) were evaluated; black spot rate (%) = (grains with spots/total grains) × 100, and control efficacy (%) = [(Grain black spot rate of control – Grain black spot rate of treatment)/Grain black spot rate of control] × 100.

##### 2.7.3.2 Insect pest efficacy assessment

Field population dynamics and control efficacy of two target rice insect pests, *N. lugens* (brown planthopper) and *C. medinalis* (rice leaf folder), were evaluated in this study. The population survey of *N. lugens* was performed following the standardized field sampling protocol described by Tyagi et al. (2022). Briefly, a five-point parallel skip sampling method was adopted, with 5 sampling points set in each plot and 10 rice hills investigated at each sampling point. The number of *N. lugens* nymphs and adults on each rice hill was counted via plant beating and visual inspection, and the average population quantity per hill was calculated for each treatment. The control efficacy against *N. lugens* was calculated using the formula: Control efficacy (%) = [(Average population quantity of untreated control - Average population quantity of treatment)/Average population quantity of untreated control] × 100. The infestation of *C. medinalis* was quantified using a two-row parallel skip sampling method, with 5 sampling points set in each plot and 2 rice hills investigated at each sampling point. The total number of leaves and the number of rolled leaves on each sampled rice hill were recorded to calculate the leaf rolling rate. The control efficacy against *C. medinalis* was calculated using the following formulas: Leaf rolling rate (%) = (Number of rolled leaves/Total number of investigated leaves) × 100; Control efficacy (%) = [(Leaf rolling rate of untreated control - Leaf rolling rate of treatment)/Leaf rolling rate of untreated control] × 100.

### 2.8 Statistical analysis

All experimental data were presented as mean ± standard error (SE) of independent biological replicates, with the number of replicates specified for each assay. All statistical analyses were performed using SPSS 26.0 software (IBM Corp., Armonk, NY, United States). For multi-group comparisons, one-way analysis of variance (ANOVA) was conducted, followed by Duncan’s new multiple range test to determine significant differences between treatments at the *p* < 0.05 significance level.

## 3 Results

### 3.1 Inhibitory efficacy of composite metabolites against rice pathogens

**FIGURE 1:**
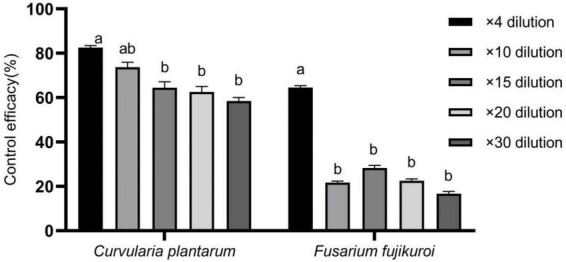
Anti-fungal effect of compound metabolic solution diluted by different folds on *C. plantarum* and *F. fujikuroi.* Data were analyzed by one-way analysis of variance (ANOVA) combined with Duncan’s method for *post-hoc* multiple comparisons. Different lowercase letters above the bars indicate significant differences among treatments (*P* < 0.05). And in this figure, significance letters are only valid for comparisons among treatments within each individual pathogen group; the letter labels are not comparable between the *Curvularia plantarum* and *Fusarium fujikuroi* groups.

*C. plantarum* and *F. fujikuroi* were selected for evaluation. As shown in Table 3, metabolites of a single *B. subtilis* strain effectively inhibited both pathogens. While TMB showed lower antimicrobial efficacy against *C. plantarum* compared to *B. subtilis* metabolites alone, its inhibition rate against *F. fujikuroi* was significantly higher (66.22% vs. 55.74%, *P* < 0.05). Further optimization revealed that increasing the proportion of *B. subtilis* metabolites in TMB formulations (TMB1–TMB4) initially enhanced but subsequently reduced inhibition of *C. plantarum* (Table 4), while the inhibition rate for *F. fujikuroi* remained stable (66.22–67.4%). This indicates pathogen-specific selectivity, with the 1:1:1 ratio providing optimal dual-pathogen suppression. Further research found that the inhibition effect of composite metabolites on pathogen was dependent on dilution, e.g., TMB maintained more than 60% inhibition of *C. plantarum* even at a 20-fold dilution, whereas the inhibition of *F. fujikuroi* dropped sharply beyond a 10-fold dilution. This further confirms the pathogen-selective activity (Figure 1).

**TABLE 3:** Inhibition effect of single and complex metabolites on different rice pathogen.

Treatment	T	M	B	TMB
*C. plantarum* inhibition rate (%)	58.78 ± 3.24b	47.27 ± 3.15b	88.72 ± 2.67a	82.66 ± 0.66a
*F. fujikuro*i inhibition rate (%)	20.09 ± 3.44c	22.82 ± 4.84c	55.74 ± 0.48b	66.22 ± 3.75a

“T” refers to metabolite of *T. asperellum*; “M” refers to metabolite of *M. anisopliae*; “B” refers to metabolite of *B. subtilis*; “TMB” refers to metabolite of *T. asperellum*, *M. anisopliae* and *B. subtilis.* Data were analyzed by one-way analysis of variance (ANOVA) combined with Duncan’s method for *post-hoc* multiple comparisons. Different lowercase letters above the bars indicate significant differences among treatments (*P* < 0.05).

**TABLE 4:** Inhibition effect of compound metabolites compounded in different proportions on rice pathogen.

Treatment	TMB	TMB1	TMB2	TMB3	TMB4
*C. plantarum* inhibition rate (%)	82.66 ± 0.66a	79.65 ± 2.56b	83.39 ± 0.59a	83.95 ± 1.04a	85.3 ± 1.18a
*F. fujikuro*i inhibition rate (%)	66.22 ± 3.75	66.33 ± 1.68	67.12 ± 1.89	66.88 ± 0.49	67.4 ± 0.61

“TMB” refers to composite microbial metabolites derived from mixtures of *T. asperellum*, *M. anisopliae*, and *B. subtilis* in varying ratios, specifically: TMB = 1:1:1, TMB1 = 1:1:2, TMB2 = 1:1:3, TMB3 = 1:1:4, TMB4 = 1:1:5.

### 3.2 Synergistic effects of TMB and chemical fungicide against rice disease

#### 3.2.1 *In vitro* pathogen inhibition

Combinations of TMB with chemical fungicides (29% trifloxystrobin⋅tebuconazole or 75% tebuconazole⋅azoxystrobin) exhibited significantly enhanced efficacy against both pathogens compared to single agents (Table 5). For instance, the combination of TMB (4-fold dilution) with 1 mg/L trifloxystrobin⋅tebuconazole inhibited *F. fujikuroi* growth by 71.89%, whereas the individual component showed 55.16–64.5% inhibition. This findings support the potential of TMB-based combinations in chemical fungicide reduction strategies.

**TABLE 5:** Inhibition rate of compound metabolic solution and chemical fungicide against *C. plantarum* and *F. fujikuroi*.

Reagent treatment		*C. plantarum* inhibition rate (%)	*F. fujikuroi* inhibition rate (%)
TMB	× 4 dilution	–	64.5 ± 0.88ab
	× 30 dilution	58.47 ± 1.58bc	–
A	1 mg/L	–	61.45 ± 3.18ab
	4 mg/L	53.53 ± 0.67c	–
C	1 mg/L	–	55.16 ± 1.62b
	8 mg/L	51.26 ± 0.26c	–
TMB+A	× 4 dilution+1 mg/L A	–	68.17 ± 2.57ab
	× 30 dilution+4 mg/L A	71.81 ± 1.54a	–
TMB+C	× 4 dilution+1 mg/L C	–	71.89 ± 2.16a
	× 30 dilution+8 mg/L C	65.3 ± 1b	–

“TMB” refers to composite microbial metabolites, with × 4 dilution and × 30 dilution indicating 4- and 30-fold diluted metabolites respectively; “A” refers to 29% trifloxystrobin-tebuconazole; “C” refers to 75% tebuconazole-pyraclostrobin; and “+” indicates combination treatment of the two agents. Data were analyzed by one-way analysis of variance (ANOVA) combined with Duncan’s method for *post-hoc* multiple comparisons. Different lowercase letters above the bars indicate significant differences among treatments (*P* < 0.05).

As shown in Table 6, the combination of reduced-dosage 29% trifloxystrobin-tebuconazole (A) with composite microbial metabolites (TMB) exhibited significantly enhanced inhibition against *C. plantarum* compared to individual applications of either agent alone (*P* < 0.05). Moreover, this combination achieved inhibition rates against *F. fujikuroi* equivalent to those of the full-dosage chemical fungicide, suggesting a synergistic interaction between reduced-dosage A with TMB. In contrast, 75% tebuconazole-azoxystrobin (C) displayed inherently high inhibitory activity, which likely masked any potential synergistic effects with TMB.

**TABLE 6:** Inhibitory rate of compound metabolic solution and reduced chemical fungicide against pathogenic fungi.

Treatment	*C. plantarum* inhibition rate (%)	*F. fujikuroi* inhibition rate (%)
TMB	82.38 ± 1.92b	63.67 ± 1.49c
TMB+A(full dose)	89 ± 0.75ab	92.32 ± 0.71a
TMB+A(-30%)	87.9 ± 0.24ab	89.9 ± 0.71a
TMB+A(-40%)	86.21 ± 0.23ab	88.15 ± 0.49a
TMB+A(-50%)	85.55 ± 0.83b	75.22 ± 3.25b
A(full dose)	88.06 ± 0.42ab	92.98 ± 0.31a
A(-30%)	75.25 ± 0.53c	90.12 ± 0.58a
A(-40%)	74.71 ± 0.97c	89.46 ± 0.44a
A(-50%)	70.67 ± 1.2c	88.81 ± 0.46a
TMB+C(full dose)	88.67 ± 0.6ab	92.76 ± 0.33a
TMB+C(-30%)	88.33 ± 0.53ab	92.76 ± 0.33a
TMB+C(-40%)	89.16 ± 0.55ab	92.76 ± 0.33a
TMB+C(-50%)	88.99 ± 0.92ab	92.76 ± 0.33a
C(full dose)	93.13 ± 0.28a	92.76 ± 0.33a
C(-30%)	93.13 ± 0.28a	92.76 ± 0.33a
C(-40%)	93.13 ± 0.28a	92.76 ± 0.33a
C(-50%)	90.79 ± 0.82ab	92.76 ± 0.33a

“TMB” denotes composite microbial metabolites; “A” represents 29% trifloxystrobin ⋅ tebuconazole; “C” indicates 75% tebuconazole azoxystrobin. The notations “-30%,” “-40%,” and “-50%” signify reductions of 30, 40, and 50% in the application rates of the respective chemical fungicides compared to full recommended field doses. Data were analyzed by one-way analysis of variance (ANOVA) combined with Duncan’s method for *post-hoc* multiple comparisons. Different lowercase letters above the bars indicate significant differences among treatments (*P* < 0.05).

#### 3.2.2 Control efficacy of biochar-based composite metabolite seed soaking agent against rice sheath blight

Pot trial results demonstrated that treatment with the biochar-based composite metabolite seed soaking agent effectively controlled rice sheath blight. Biochar-based TMB exhibited superior efficacy, achieving 76.56 ± 1.59% disease control. Individual treatments with either *T. asperellum* metabolites or *M. anisopliae* showed comparable control efficacy against rice sheath blight, both exceeding 65% (Table 7).

**TABLE 7:** Control efficacy of seed impregnation on rice sheath blight.

Seed soaking	Plants investigated (n)	Disease index	Control efficacy (%)
T	141	10.92 ± 1.24cb	68.69 ± 3.49bc
M	133	11.88 ± 1.35bc	65.94 ± 3.84bc
B	97	14.32 ± 0.94b	58.62 ± 2.68c
TMB	115	8.17 ± 0.56c	76.56 ± 1.59a
CK	125	34.88 ± 2.16a	–

Data were analyzed by one-way analysis of variance (ANOVA) combined with Duncan’s method for *post-hoc* multiple comparisons. Different lowercase letters above the bars indicate significant differences among treatments (*P* < 0.05).

#### 3.2.3 Effects of biochar-based TMB seed coating on pathogen suppression and rice growth

Seed treatment with single metabolites revealed that *T. asperellum* (T) and *M. anisopliae* (M) metabolites enhanced overall seedling growth, while T and *B. subtilis* (B) metabolites specifically promoted germ and radicle development (Tables 8–10). For radicle elongation, preventive treatment (metabolite immersion followed by pathogen exposure) exhibited significantly higher effects than curative treatment (pathogen exposure followed by metabolites) (Table 9). Conversely, germ growth was preferentially enhanced by preventive treatment under *C. plantarum* challenge but by curative treatment under *F. fujikuroi* infection. Critically, regardless of treatment sequence, the composite metabolite TMB (1:1:1 ratio of T:M:B) consistently delivered significantly higher germination rates, radicle lengths, and germ lengths compared to both single metabolites and controls.

**TABLE 8:** The effect of the sequential treatment of composite microbial metabolic solution and pathogenic fungi on rice seeds germination rate.

Treatment^a^	T	M	B	TMB	CK
Metabolite+*C. plantarum*	43.33 ± 3.33b	58.89 ± 1.92a	42.22 ± 3.85b	63.33 ± 8.82a	60 ± 3.33a
*C. plantarum*+Metabolite	60 ± 3.33a	60 ± 3.33a	56.67 ± 3.33ab	64.44 ± 6.94a	50 ± 3.33b
Metabolite+*F. fujikuroi*	71.11 ± 5.09a	46.67 ± 3.33b	47.78 ± 1.92b	62.22 ± 10.18a	44.44 ± 1.92b
*F. fujikuroi*+Metabolite	56.67 ± 3.33a	48.89 ± 1.92b	46.67 ± 3.33b	61.11 ± 5.09a	40 ± 3.33c

*^a^*The notation “metabolites + pathogen” denotes seed treatment with microbial metabolites for 24 h followed by pathogen spore suspension exposure for 24 h (preventive protocol). Conversely, “pathogen + metabolites” indicates initial exposure to pathogen spores for 24 h prior to 24 h metabolite treatment (curative protocol). Data were analyzed by one-way analysis of variance (ANOVA) combined with Duncan’s method for *post-hoc* multiple comparisons. Different lowercase letters above the bars indicate significant differences among treatments (*P* < 0.05).

**TABLE 9:** The effect of the sequential treatment of composite microbial metabolic solution and pathogenic fungi on rice radicle growth.

Treatment^a^	T	M	B	TMB	CK
Metabolite+*C. plantarum*	5.98 ± 1.24a	5.76 ± 0.59b	6.01 ± 1.38a	6.12 ± 0.56a	4.76 ± 0.98c
*C. plantarum*+Metabolite	4.99 ± 0.99ab	4.86 ± 0.57b	5.01 ± 0.66ab	5.11 ± 0.95a	4.65 ± 1.25b
Metabolite+*F. fujikuroi*	5.48 ± 1.23	5.37 ± 2.11	5.46 ± 1.26	5.67 ± 0.56	5.22 ± 0.98
*F. fujikuroi*+Metabolite	5.31 ± 0.59	5.21 ± 0.95	5.12 ± 0.13	5.54 ± 0.22	5.32 ± 0.49

*^a^*The notation “metabolites + pathogen” denotes seed treatment with microbial metabolites for 24 h followed by pathogen spore suspension exposure for 24 h (preventive protocol). Conversely, “pathogen + metabolites” indicates initial exposure to pathogen spores for 24 h prior to 24 h metabolite treatment (curative protocol). Data were analyzed by one-way analysis of variance (ANOVA) combined with Duncan’s method for *post-hoc* multiple comparisons. Different lowercase letters above the bars indicate significant differences among treatments (*P* < 0.05).

**TABLE 10:** The effect of the sequential treatment of composite microbial metabolic solution and pathogenic fungi on rice germ growth.

Treatment^a^	T	M	B	TMB	CK
Metabolite+*C. plantarum*	3.19 ± 0.13ab	3.06 ± 0.05ab	2.92 ± 0.36ab	3.61 ± 0.06a	2.57 ± 0.11b
*C. plantarum*+Metabolite	2.82 ± 0.19	2.61 ± 0.12	2.69 ± 0.18	2.94 ± 0.2	2.57 ± 0.11
Metabolite+*F. fujikuroi*	3.1 ± 0.31ab	2.93 ± 0.48b	2.91 ± 0.29b	3.42 ± 0.11a	2.52 ± 0.26c
*F. fujikuroi*+Metabolite	3.43 ± 0.45c	2.98 ± 0.18d	3.73 ± 0.28b	3.76 ± 0.49a	2.88 ± 0.27e

*^a^*The notation “metabolites + pathogen” denotes seed treatment with microbial metabolites for 24 h followed by pathogen spore suspension exposure for 24 h (preventive protocol). Conversely, “pathogen + metabolites” indicates initial exposure to pathogen spores for 24 h prior to 24 h metabolite treatment (curative protocol). Data were analyzed by one-way analysis of variance (ANOVA) combined with Duncan’s method for *post-hoc* multiple comparisons. Different lowercase letters above the bars indicate significant differences among treatments (*P* < 0.05).

### 3.3 Field application of composite metabolites TMB with reduced chemicals for rice pest and disease control

#### 3.3.1 Rice sheath blight control

**FIGURE 2:**
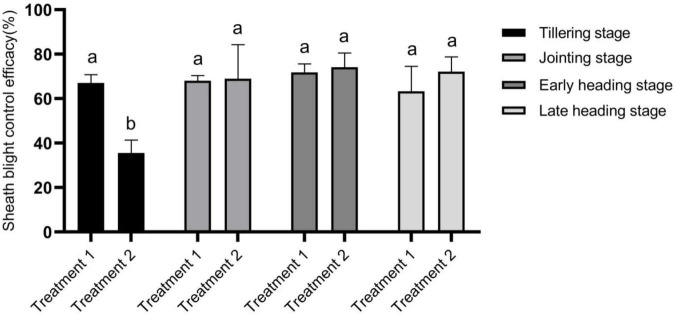
Control efficacy of combined metabolites TMB on Rice Sheath Blight in different growth periods in Jinshan District, Shanghai. The treatments in the figure indicate: Treatment 1: TMB + 40% reduced chemical pesticides.

**FIGURE 3:**
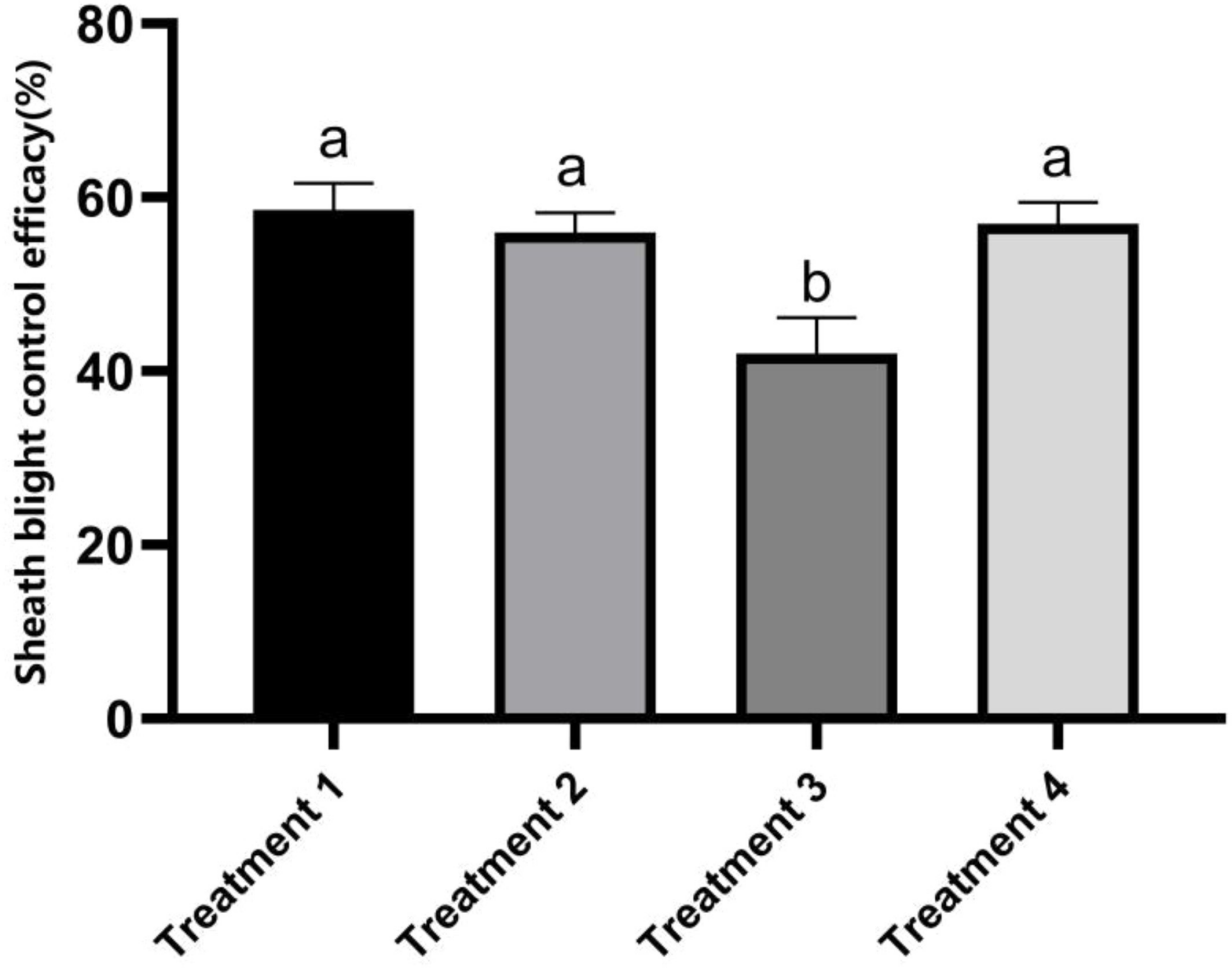
Control efficacy of combined metabolites TMB on Rice Sheath Blight in different growth periods in Songjiang District, Shanghai. Treatment 1: TMB + 30% reduced 29% trifloxystrobin⋅tebuconazole; Treatment 2: TMB + 30% reduced 75% azoxystrobin⋅tebuconazole; Treatment 3: Full-dose 29% trifloxystrobin⋅tebuconazole; Treatment 4: Full-dose 29% trifloxystrobin⋅tebuconazole. Data were analyzed by one-way analysis of variance (ANOVA) combined with Duncan’s method for *post-hoc* multiple comparisons. Different lowercase letters above the bars indicate significant differences among treatments (*P* < 0.05).

At the Jinshan District site (Figure 2), foliar application of TMB + 40% reduced chemicals during tillering achieved 57.06% control efficacy against sheath blight (*R. solani*), surpassing full-dose chemical treatment (66.72% > 35.55%, *P* < 0.05). From the jointing stage onward, the control efficacy of the TMB + reduced chemicals treatment became comparable to that of the full-dose chemicals treatment, indicating a stronger synergistic effect during the early growth stages. The 40% chemical reduction may exceed the optimal threshold, suggesting 10–20% reduction could enhance synergy with TMB. At the Songjiang District site (Figure 3), TMB combined with 30% reduced fungicides (29% trifloxystrobin⋅tebuconazole or 75% tebuconazole⋅azoxystrobin) at booting stage outperformed full-chemical treatments (e.g., 89.7% vs 82.1%), demonstrating the composite metabolites’ capacity to replace partial chemical inputs while maintaining or improving efficacy.

At the Songjiang trial site, efficacy assessment against Rice Sheath Blight was exclusively conducted during the booting stage. Results demonstrated that combining TMB with 30% reduced chemical pesticides consistently achieved superior control efficacy compared to full-dose chemical treatments alone (Figure 3). This indicates that the composite microbial metabolites can effectively substitute for a portion of chemical inputs while delivering comparable or even enhanced disease suppression.

#### 3.3.2 Rice spikelet rot disease control

**FIGURE 4:**
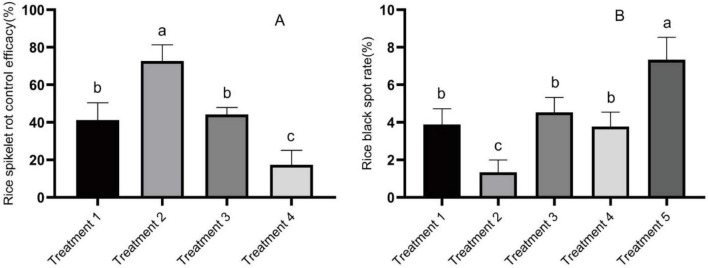
Control efficacy of composite metabolite TMB against Rice Spikelet Rot disease **(A)** and Rice Black Spot **(B)** in Songjiang test site, Shanghai. Treatment 1: TMB + 30% reduced 29% trifloxystrobin-tebuconazole; Treatment 2: TMB + 30% reduced 75% tebuconazole ⋅ azoxystrobin; Treatment 3: Full-dose 29% trifloxystrobin-tebuconazole; Treatment 4: Full-dose 75% tebuconazole ⋅ azoxystrobin; Treatment 5: Full-dose conventional chemical pesticides for production fields. Data were analyzed by one-way analysis of variance (ANOVA) combined with Duncan’s method for *post-hoc* multiple comparisons. Different lowercase letters above the bars indicate significant differences among treatments (*P* < 0.05).

**FIGURE 5:**
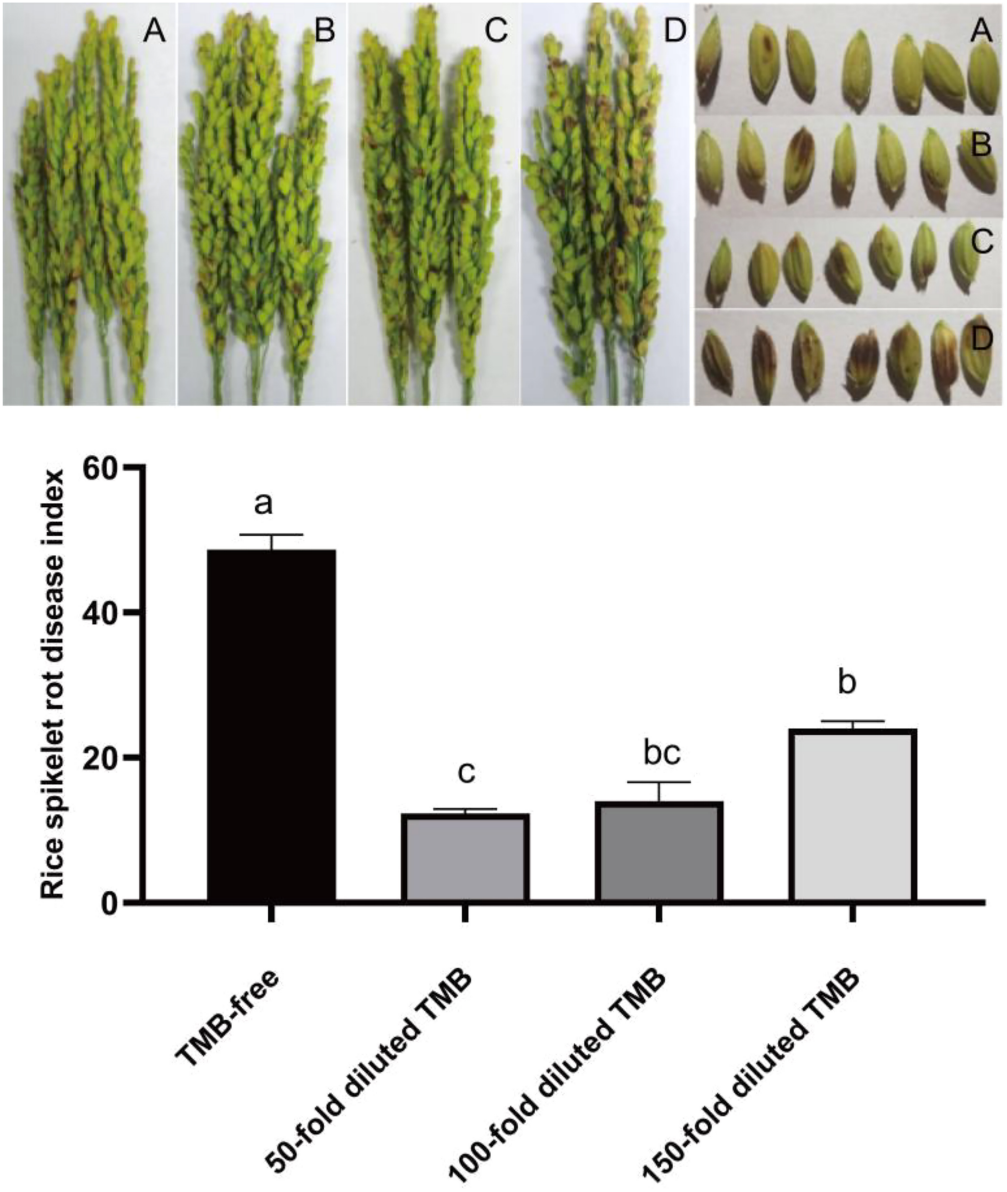
Control efficacy of different concentrations of composite metabolite TMB on Rice Spikelet Rot Disease in Jinshan District, Shanghai. A: 150 × diluted TMB; B: 100 × diluted TMB; C: 50 × diluted TMB; D: Control without TMB. Data were analyzed by one-way analysis of variance (ANOVA) combined with Duncan’s method for *post-hoc* multiple comparisons. Different lowercase letters above the bars indicate significant differences among treatments (*P* < 0.05).

**FIGURE 6:**
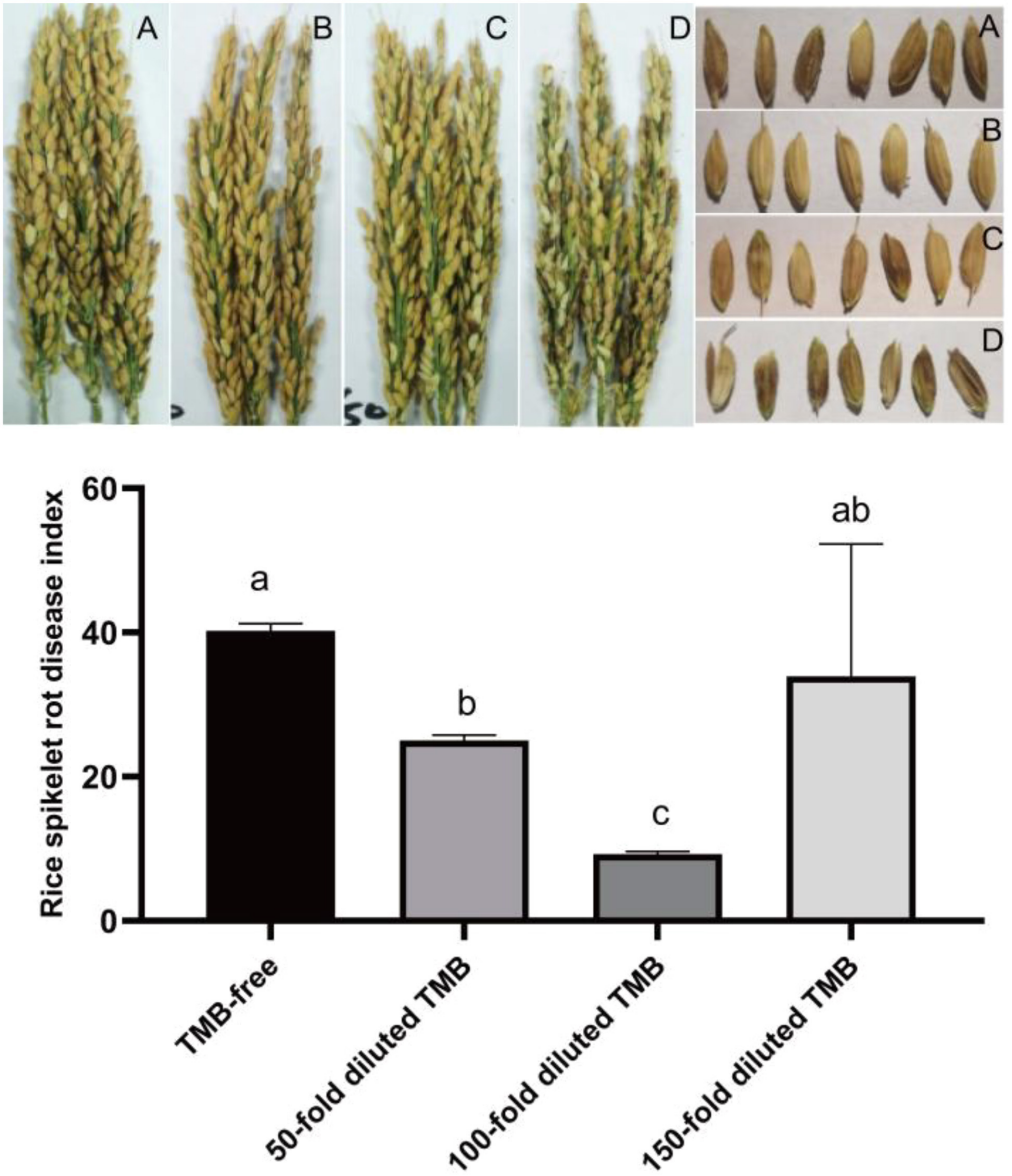
Control Efficacy of Differently Diluted Composite Metabolite TMB against Rice Spikelet Rot Disease in Songjiang District, Shanghai. A: 150 × diluted TMB; B: 100 × diluted TMB; C: 50 × diluted TMB; D: Control without TMB. Data were analyzed by one-way analysis of variance (ANOVA) combined with Duncan’s method for *post-hoc* multiple comparisons. Different lowercase letters above the bars indicate significant differences among treatments (*P* < 0.05).

As shown in Figure 4, the combined application of the composite metabolite TMB with 75% tebuconazole⋅azoxystrobin at 30% reduced dose resulted in significantly superior control efficacy against rice spikelet rot compared to other treatments. Therefore, this demonstrates that the composite metabolite TMB also exhibits notable efficacy against rice spikelet rot. Furthermore, it effectively reduces the incidence of rice grain black spot. In addition, we investigated the effect of foliar application of the composite metabolite TMB alone (without chemical pesticides) at different dilution ratios on the incidence of rice spikelet rot disease. The results from the Jinshan and Songjiang districts showed that the disease index of rice spikelet rot disease was the lowest when TMB was diluted 100-fold (Figures 5, 6).

#### 3.3.3 Brown planthopper control

**FIGURE 7:**
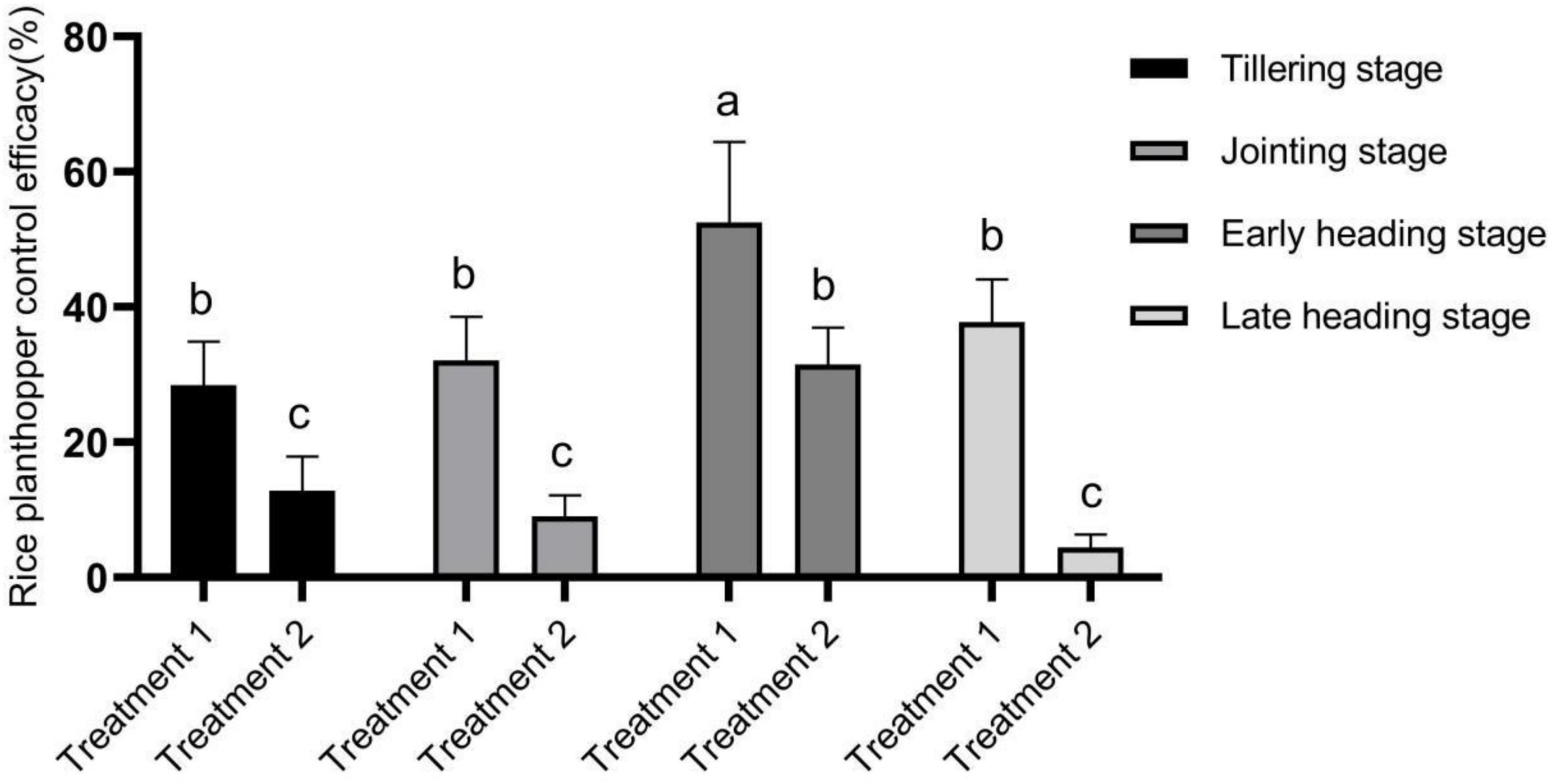
The control efficacy of different treatments against brown planthopper (*N. lugens*) at different stages in Jinshan District, Shanghai. Treatment 1: TMB + 40% reduced chemical pesticides; Treatment 2: Full-dose chemical pesticides. Data were analyzed by one-way analysis of variance (ANOVA) combined with Duncan’s method for *post-hoc* multiple comparisons. Different lowercase letters above the bars indicate significant differences among treatments (*P* < 0.05).

The results showed that at the Jinshan trial site, the TMB + 40% reduced chemical pesticide combination consistently maintained higher efficacy in controlling the brown planthopper (*N. lugens*) across all rice growth stages, achieving approximately 50% efficacy during early heading stage (Figure 7). These findings confirm that robust performance of TMB in brown planthopper management and its compatibility with reduced-chemical integrated strategies.

#### 3.3.4 Rice leaf roller control

**FIGURE 8:**
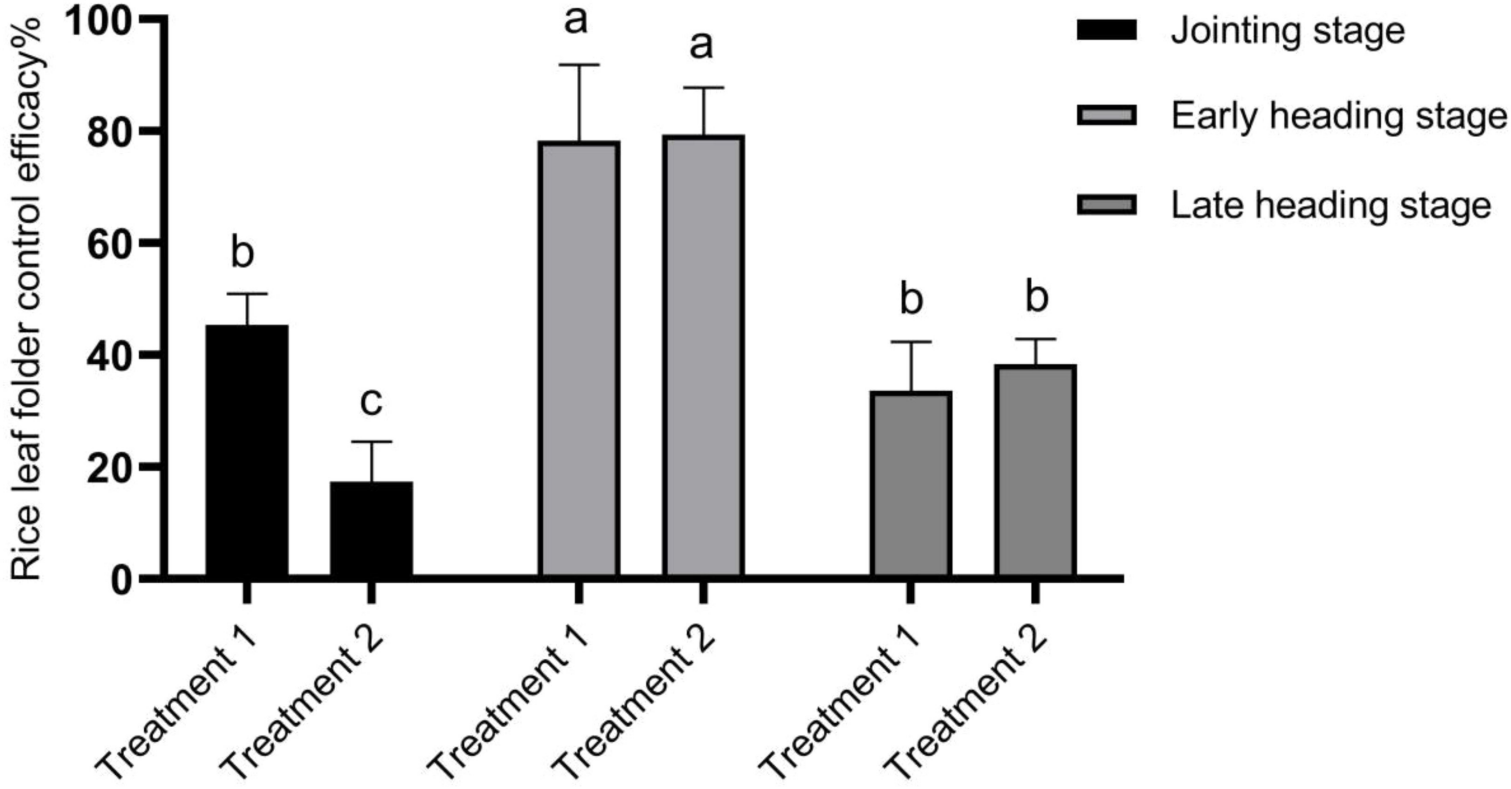
Control efficacy of different treatments on rice leaf roller (*C. medinalis*) in different periods in Jinshan District, Shanghai. Treatment 1: TMB + 40% reduced chemical pesticides; Treatment 2: Full-dose chemical pesticides (29% trifloxystrobin⋅tebuconazole). Data were analyzed by one-way analysis of variance (ANOVA) combined with Duncan’s method for *post-hoc* multiple comparisons. Different lowercase letters above the bars indicate significant differences among treatments (*P* < 0.05).

The control efficacy of different treatments against *C. medinalis* exhibited distinct stage-specific patterns at the Jinshan District trial site (Figure 8). At the jointing stage, Treatment 1 (TMB combined with 40% reduced-dosage chemical pesticides) achieved a mean control efficacy of 45.41%, which was significantly higher than the 17.38% efficacy of Treatment 2 (full-dose chemical pesticides) (*P* < 0.05). In contrast, at both the early and late heading stages, the two treatments showed comparable control efficacy. These results indicate that the synergistic system of TMB and chemical pesticides provides significantly stronger control against *C. medinalis* during the early growth stages of rice compared with conventional full-dose chemical application.

### 3.4 Preliminary study on the active ingredients and stability of the biochar-based TMB composite metabolite

**FIGURE 9:**
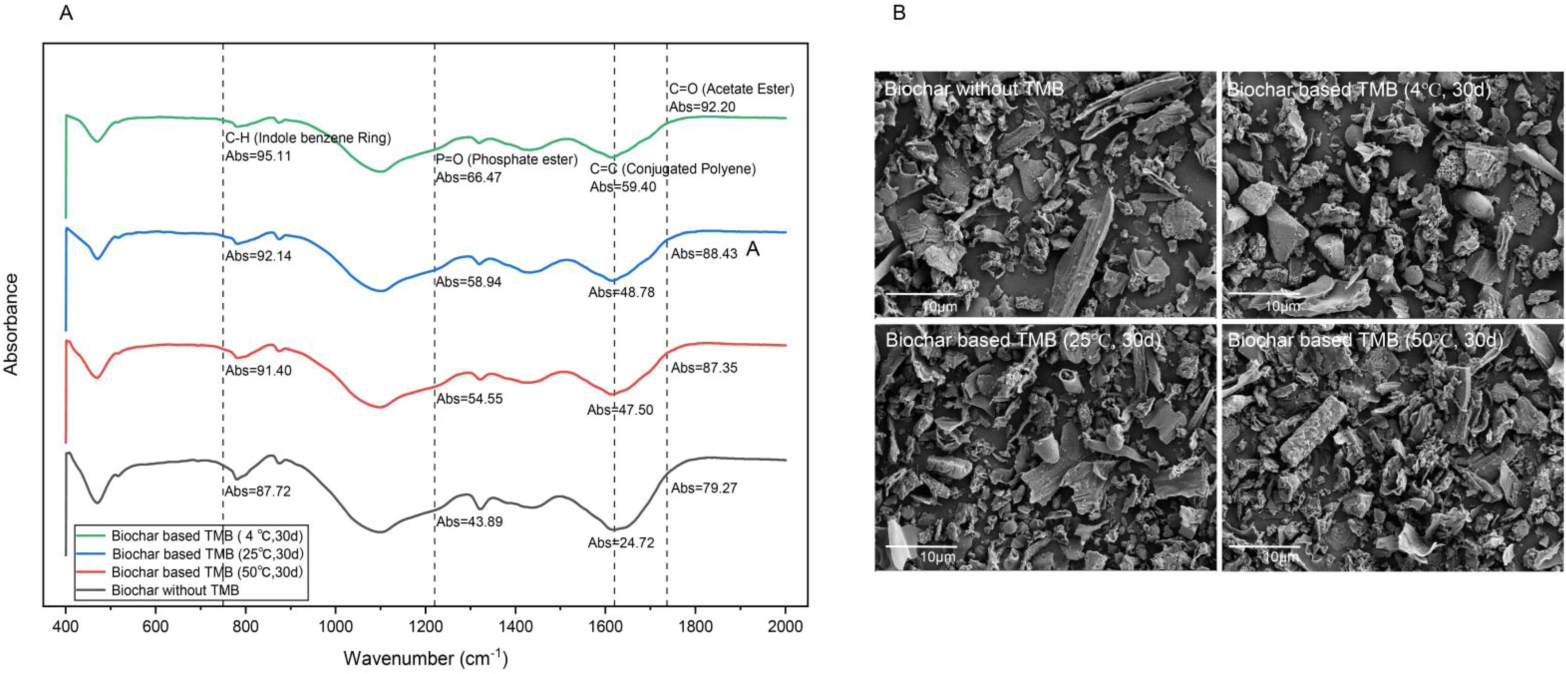
Adsorption performance of biochar on TMB composite metabolites. **(A)** Absorbance corresponding to the functional groups of the compounds in TMB composite metabolites mixed with biochar after 30 days of storage at different temperatures. **(B)** Scanning electron microscopy (SEM) images of TMB composite metabolites mixed with biochar after 30 days of storage at different temperatures.

**FIGURE 10:**
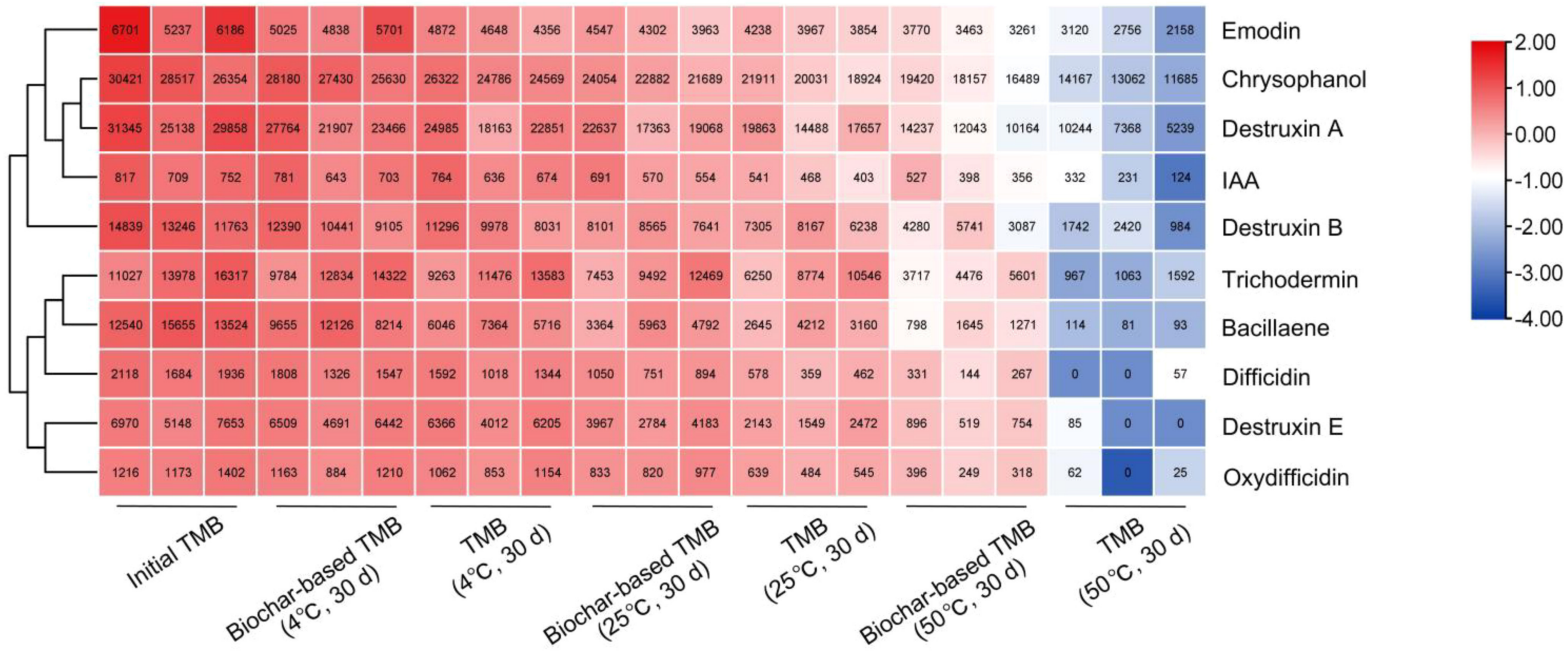
Changes in the relative contents of partial marker compounds in the biochar-based TMB composite metabolite after 30 days of storage at different temperatures.

Given the favorable control efficacy of the biochar-based TMB seed coating agent against rice diseases described above, we further conducted a preliminary investigation into the bioactive components in TMB and the adsorption performance of biochar for these active components. We first detected a panel of bioactive components in the fermentation broths of *T. asperellum*, *M. anisopliae*, and *B. subtilis*, respectively, whose bioactivities covered antifungal, antibacterial, insecticidal, and plant growth-regulating effects (Table 11). Subsequently, Fourier transform infrared spectroscopy (FTIR) was performed to determine the absorbance values of characteristic functional groups of organic compounds in the biochar-TMB mixture after 30 days of storage at different temperatures (Figure 9A). At 4 °C and room temperature, the absorbance values of these characteristic functional groups were all higher than those of the biochar control without TMB supplementation, indicating that biochar adsorbed a portion of the compounds from TMB. Notably, the absorbance values of these characteristic functional groups remained higher than those of the TMB-free biochar control even after the biochar-amended TMB was stored at 50 °C for 30 days. We therefore hypothesized that the adsorption and encapsulation capacity of biochar protected these bioactive compounds, thereby reducing their degradation rate. This hypothesis was corroborated by the compound detection results via high-performance liquid chromatography-tandem mass spectrometry (HPLC-MS) (Figure 10): the 10 bioactive compounds listed in Table 11 were still detectable in biochar-amended TMB after 30 days of storage, whereas they were barely detectable in TMB without biochar addition. Furthermore, there were no significant differences in the morphological structures of TMB composite metabolites mixed with biochar even after being stored at 4–50°C for 30 days by scanning electron microscopy (SEM) images (Figure 9B).

**TABLE 11:** Partial functional compounds derived from *T. asperellum*, *M. anisopliae* and *B. subtilis* detected in the TMB composite metabolite.

Source	Component name	Retention TIME (min)	Observed m/z	Function
T	Emodin	4.97	271.0610	Anti-fungal/bacterial activity (Zhan et al., 2024)
T	Chrysophanol	4.20	255.0657	Anti-fungal/bacterial activity (Liu et al., 2016)
T	Trichodermin	4.59	275.1624	Anti-fungal activity (Chen et al., 2021)
M	Destruxin A	6.44	578.3545	Insectic idal activity (Kiruthiga et al., 2026)
M	Destruxin B	7.90	594.3859	Insecticidal activity (Wu et al., 2013)
M	Destruxin E	5.22	594.3500	Insecticidal activity (Yeh et al., 1996)
B	Bacillaene	10.19	613.3933	Anti-bacterial activity (Miao et al., 2024)
B	Difficidin	11.00	567.2820	Anti-bacterial activity (Wu et al., 2015)
B	Oxydifficidin	9.81	559.2822	Anti-bacterial activity (Chernyshova et al., 2025)
T, M and B	IAA	3.64	174.0560	Plant growth promoter (Hayashi et al., 2021)

T, *T. asperellum*; M, *M. anisopliae*; B, *B. subtilis*.

## 4 Discussion

Beneficial microbial secondary metabolites have emerged as a core pillar of green plant protection technology, with inherent advantages including extended shelf stability, rapid onset of biocontrol activity, and high compatibility with conventional chemical pesticides, which overcome the critical limitations of live microbial biocontrol agents in field application (Besset-Manzoni et al., 2018; Paege et al., 2024). However, most existing metabolite-based biocontrol products rely on single-strain metabolites, which suffer from a narrow pest control spectrum and fail to address the complex disease-insect pest complexes prevalent in rice production systems. To fill this gap, we developed a ternary composite metabolite system (TMB) integrating functional metabolites from *T. asperellum*, *M. anisopliae*, and *B. subtilis*, with complementary antifungal, insecticidal, and plant growth-promoting functions. While synergies between individual biocontrol agents and chemical pesticides in rice pest management have been previously documented (Zhang et al., 2021), this study is the first to establish a synergistic application system combining a broad-spectrum ternary microbial metabolite composite with reduced-dosage chemical pesticides, providing an innovative model for sustainable rice health management.

Compared with synthetic chemical pesticides that act on specific single molecular targets (Matsuda et al., 2020; Yu et al., 2023), microbial biocontrol metabolites exert their activity through multiple action sites and modes, including direct antagonism against pathogens and insect pests, and induction of systemic resistance in host plants. This multi-target mode of action not only reduces the selective pressure of chemicals on pathogens and pests, but also effectively delays the development of pesticide resistance (Shahriar et al., 2022). The synergistic enhancement of control efficacy achieved by the combination of TMB and reduced-dosage chemical pesticides in our study can be explained by three potential mechanisms: first, reduced chemical dosage alleviates the inhibitory effect of synthetic fungicides on the functional activity of microbial metabolites; second, chemical pesticides weaken the vitality and defense capacity of pathogens and insect pests, making them more susceptible to microbial metabolites; third, TMB enhances the systemic resistance of rice plants to diseases and insect pests, which complements the direct toxic effect of chemical pesticides and achieves comprehensive control efficacy. These findings align with previous studies showing that combinations of microbial metabolites and chemical pesticides can achieve complementary advantages and synergistic control effects against agricultural pests (Zhang et al., 2025; Ayaz et al., 2024).

The biochar-based TMB seed coating agent developed in this study showed excellent performance in suppressing seed-borne pathogens and alleviating pathogen-induced growth inhibition in rice seedlings. We confirmed that biochar is well compatible with TMB, and has great potential as an adjuvant for TMB to improve the stability of active metabolites. In future work, we will also further improve the shelf-life evaluation of the biochar-based TMB formulation. The enhanced biocontrol efficacy and plant growth-promoting effect of the biochar-based formulation may be attributed to multiple synergistic mechanisms: previous studies have demonstrated that biochar can optimize the rhizosphere microenvironment by improving soil nutrient availability, regulating rhizosphere microbial community structure, and suppressing the proliferation of soil-borne pathogens (Li et al., 2024; Wu et al., 2025). Meanwhile, the porous structure of biochar enables the adsorption and sustained release of bioactive metabolites, which protects the active components from degradation, extends the effective period of the formulation, and enhances its biocontrol performance (Chen X. et al., 2025; Hao et al., 2025; Wang et al., 2022).

A critical concern regarding broad-spectrum biocontrol agents is their potential impact on non-target organisms and agroecosystem health. Unlike broad-spectrum chemical pesticides that cause non-target toxicity to beneficial soil microbiota, natural enemies of pests, and aquatic organisms (Yadav et al., 2015; Md Meftaul et al., 2020; Narayanan et al., 2024), the TMB formulation developed in this study is composed of naturally derived microbial secondary metabolites, which are inherently biodegradable and have low environmental persistence. The active components identified in TMB, including antifungal, insecticidal and plant growth-regulating compounds, are naturally produced by widely used beneficial microorganisms, and previous studies have confirmed their high selectivity to target pathogens and insect pests with minimal adverse effects on non-target organisms (Kaspar et al., 2019; Zin and Badaluddin, 2020; Beys-da-Silva et al., 2020). In addition, the synergistic application system of TMB with reduced-dosage chemical pesticides achieved a 30% reduction in chemical pesticide inputs in rice production, which directly reduces the environmental pollution and ecological risks caused by excessive chemical application, and is conducive to maintaining the biodiversity and intrinsic pest regulation capacity of farmland ecosystems. Subsequently, we will further systematically conduct the biosafety evaluation of this application system on non-target beneficial organisms.

This study is our team’s foundational step in developing a new generation of metabolite-based green pesticides. We have preliminarily identified multiple functional bioactive components in TMB, but the specific metabolites responsible for the core biocontrol efficacy, as well as their synergistic interaction mechanisms, remain to be further clarified. Our subsequent basic research will focus on the systematic isolation and identification of key functional metabolites in the TMB system, verification of their independent and synergistic biocontrol activities, and exploration of their molecular mechanisms of action, to provide a solid theoretical basis for the rational optimization of the TMB formulation.

Collectively, this study establishes an innovative synergistic pest management strategy using a ternary composite microbial metabolite system combined with substantially reduced chemical pesticide inputs. This integrated system not only achieves efficient control of major rice diseases and insect pests, but also realizes a significant reduction in chemical pesticide inputs in rice production, providing a robust scientific basis and feasible technical solution for the development of green, efficient, and sustainable rice health management strategies.

## Data Availability

The original contributions presented in this study are included in the article/supplementary material, further inquiries can be directed to the corresponding authors.
